# Comparative effectiveness of intrathecal morphine versus erector spinae plane block for analgesia after scoliosis surgery: a retrospective analysis

**DOI:** 10.1016/j.bas.2026.105957

**Published:** 2026-02-02

**Authors:** Alexis Raynaud, Fanny Planquart, Julien Pottecher, Yann Philippe Charles

**Affiliations:** a Service D’Anesthésie-Réanimation et Médecine Périopératoire, Hôpitaux Universitaires de Strasbourg, 1 Avenue Molière, Strasbourg, France; b Service de Chirurgie Du Rachis, Hôpitaux Universitaires de Strasbourg, 1 Avenue Molière, Strasbourg, France; c Université de Strasbourg, 4 Rue Blaise Pascal, Strasbourg, France

**Keywords:** Idiopathic scoliosis, Perioperative analgesia, Intrathecal morphine, Erector spinae plane block

## Abstract

**Objective:**

To compare the efficacy of analgesia techniques after idiopathic scoliosis surgery.

**Methods:**

Register data of scoliosis patients were analyzed between January 2020 and June 2023. Patients were divided into four groups: intravenous anesthesia (IA) only, Erector Spinae Plane Block (ESPB), Intra-Thecal Morphine (ITM) and association of ESPB + ITM. The primary outcome measure was morphine consumption median [interquartile range] at 24 h postoperatively. We also investigated morphine consumption at 48 and 72 h, pain scores, co-analgesic drugs, and specific complications: respiratory, cardiovascular, post-lumbar puncture syndrome.

**Results:**

Among 119 patients, 52 (43.7%) received IA, 32 ESPB (26.9%), 9 ITM (7.6%) and 26 (21.8%) ITM + ESPB. Morphine consumption at 24 h was significantly (p < 0.001) reduced for patients with ITM: IA 51 mg (38–71), ESPB 46 mg (30–70), ITM 12 mg (7–29), ITM + ESPB 20 mg (10–28). This superior effect of ITM was sustained until 72 h for morphine consumption and pain scores. Compared to other techniques, the visual analog scale for pain assessment revealed a significant (p < 0.001) reduction by more than 2 points when using ITM within the first 24 postoperative hours, and lower median sufentanil doses were required during anesthesia. No postoperative complications occurred.

**Conclusion:**

In this retrospective cohort, ITM was associated with significantly reduced opioid use and improved pain scores. ESPB did not show a significant additive effect. These findings should be interpreted with caution due to the retrospective design and potential selection bias. Prospective randomized trials are needed to confirm these results.

**Level of evidence:**

3 - Retrospective study.

## Highlights

• Intrathecal morphine (100 μg) significantly improves early postoperative pain control and reduces opioid consumption.
• Low-dose intrathecal morphine showed a strong safety profile with no adverse events such as respiratory depression.
• In this study Erector Spinae Plane Block (ESPB) showed no additional analgesic benefit when combined with intrathecal morphine.
• Intrathecal morphine is a key option for Enhanced Recovery After Surgery (ERAS) protocols in scoliosis surgery.

## 1 Introduction

Adolescent idiopathic scoliosis (AIS) correction and fusion are extensive procedures that require effective pain management (Borgeat and Blumenthal, 2008). Poorly controlled pain and high postoperative opioid consumption can delay mobilization, increase the risk of complications and lead to longer hospital stays with increased costs (Lachiewicz, 2013). Multimodal analgesia is recommended when following Enhanced Recovery After Surgery (ERAS) principles (Gornitzky et al., 2016; Poon et al., 2023). This approach combines different drugs and techniques such as regional anesthesia (RA) (Dunn et al., 2016).

The Erector Spinae Plane Block (ESPB) is a RA technique that has been introduced in 2016 and gained increasing interest (Forero et al., 2016). Injecting a local anesthetic between the erector spinae muscle and the transverse process of the vertebra provides multilevel analgesia. By blocking the dorsal rami of spinal nerves, the ESPB reduces the need for intravenous analgesics and supports postoperative recovery (Ma et al., 2021). While initial studies were encouraging, subsequent results of prospective remain inconsistent, highlighting the need for further research to confirm its role in scoliosis surgery (Ma et al., 2021; Oh et al., 2022; Akesen et al., 2022).

Intra-Thecal Morphine (ITM) has been used for decades as an effective perioperative analgesic in major spine surgeries (Wang et al., 1979; Pendi et al., 2017). Morphine's hydrophilic property extends its analgesic effect up to 24 h after intrathecal injection by strongly binding to central nervous system opioid receptors (Bujedo, 2014; Hindle, 2008). Despite its established analgesic benefits, regular use of ITM is often limited by concerns about respiratory depression, nausea, pruritus, and sedation (Raffaeli et al., 2006; Rawal, 2023a). While ITM is cost-effective and may promote early recovery, uncertainties regarding optimal dosing and side effect management persist, requiring careful patient selection and monitoring (France et al., 1997; Gehling and Tryba, 2009; Gonvers et al., 2021; Rawal, 2023b).

Our center provides surgery for AIS and analgesic techniques (including ESPB and ITM) were introduced within the last five years. We hypothesized that the combination of ESPB and ITM could be beneficial for opioid-sparing based on their distinct mechanisms of action involving both central and peripheral pathways, potentially offering pharmacodynamic synergy and improving postoperative pain management in AIS surgery. By evaluating their performance, we aimed at providing data that could refine ERAS protocols.

The purpose of this study was to analyze the analgesic efficacy of ESPB and ITM while determining the role of each technique in lowering 24 h postoperative morphine consumption as primary outcome parameter. Secondary objectives were the analysis of intraoperative anesthetics, postoperative Visual Analog Scale (VAS) for pain, the influence of patient- and surgery-related factors and safety. A retrospective design was chosen to evaluate the real-world efficacy of these protocols in our institution and to generate preliminary data for future prospective trials.

## 2 Materials and methods

The Scoliosis Analgesia Per-Operative Strategies (SAPOS) study was a retrospective, monocentric, and non-interventional study conducted to compare different perioperative analgesic strategies in scoliosis surgery. The study analyzed medical records of patients who underwent instrumented scoliosis correction at a specialized spine center between January 1, 2020, and June 30, 2023. The study was approved by the Ethics Committee (CE-2023-62). Our study follows the Strengthening the Reporting of Observational Studies in Epidemiology (STROBE) Statement guidelines for reporting observational studies and was declared on ClinicalTrials.gov ID NCT06194279. Patients were included using a consecutive sampling method. All patients over 15 years of age who underwent surgical correction of idiopathic scoliosis were included.

Exclusion criteria were previous history of spinal surgery (as this could alter RA effectiveness), daily use of opioids (due to risks of tolerance and hyperalgesia), opposition from patients or their legal guardians to collect their data and legal protection measures.

The primary outcome measure was total morphine consumption during the first 24 postoperative hours, including intravenous morphine titration in the recovery room administered by the nurse or anesthesiologist, morphine doses administered as patient-controlled analgesia (PCA) and oral morphine intake in the surgical unit. All doses were standardized into intravenous morphine equivalents and expressed as medians with interquartile ranges to minimize the impact of extreme values. Secondary outcomes included morphine consumption at 48 and 72 h postoperatively, intraoperative sufentanil consumption, postoperative pain (VAS) assessment, functional recovery metrics such as time to first mobilization and length of hospital stay. Postoperative adverse events and specific complications for RA and morphine administration were recorded: respiratory, cardiovascular, post-lumbar puncture syndrome.

Patients were divided into four groups: those who received intravenous anesthesia only (*IA*), those who underwent ESPB alone (*ESPB*), those who received ITM alone (*ITM*), and those who were treated with a combination of ITM and ESPB (*ESPB + ITM*).

**Fig. 1:**
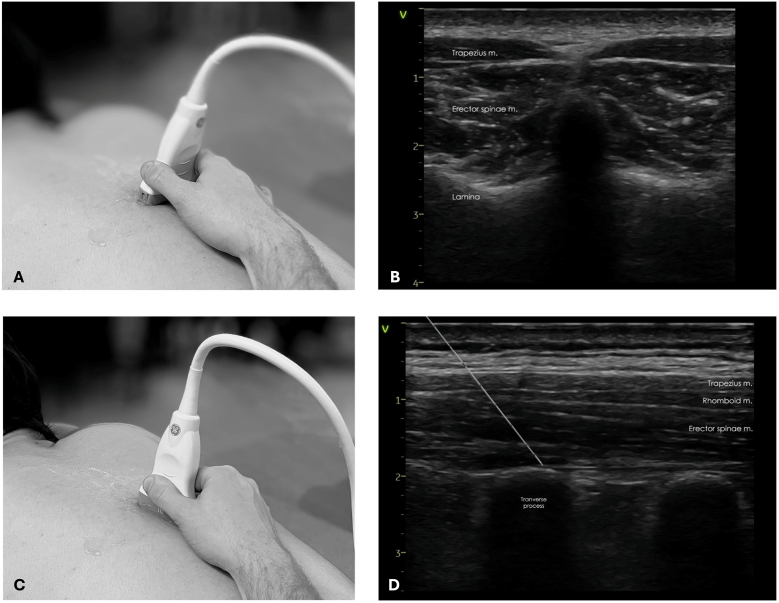
Ultrasound-guided localization for Erector Spinae Plane Block (ESPB): transverse ultrasound positioning (A) and view of the thoracic vertebra (B), longitudinal ultrasound positioning (C), view of the thoracic transverse processes and target trajectory (D).

The ESPB was performed under ultrasound guidance, targeting the fascia between the transverse process and the erector spinae muscles (Fig. 1). Ropivacaine was injected bilaterally at several levels. The number of levels and the volume injected depended on the scoliosis type and fusion length. The concentration of ropivacaine (0.1% or 0.2%) was determined by the anesthesiologist in charge and doses ranged between 40 mg and 80 mg remaining under the recommended safe limit of total local anesthetic dose, depending on the patient's weight (Yelnik et al., 2019).

For ITM, a morphine dose corresponding to 1–2 μg/kg according to patient's weight (median dose 100 μg) was administered intrathecally the same way as recommended for major spine surgery (Wang et al., 1979; Pendi et al., 2017). The injection was performed before anesthesia induction by the anesthesiologist and was usually performed at the lumbar levels L3-L4 or L4-L5. General anesthesia was provided with total intravenous anesthesia. Devices for intraoperative nociception assessment were not used during surgery and subsequent intraoperative sufentanil injections were decided based on the anesthesiologist's assessment.

Preoperative data included demographic and clinical parameters such as age, sex, body mass index (BMI), American Society of Anesthesiology (ASA) score, scoliosis classification based on the Lenke classification (Lenke et al., 2001). Perioperative data included the number of vertebrae fused, duration of surgery, usage of other analgesic drugs as well as VAS, morphine consumption, oxygen requirement, time to first mobilization, time to urinary catheter removal, return of bowel function, length of hospital stay, and complications.

Data were analyzed using R Software (Version 4.1.1, R Foundation for Statistical Computing, Vienna, Austria). Continuous variables were expressed as medians with interquartile ranges, while categorical variables were presented as counts and percentages. The Kruskal-Wallis test was used for group comparisons, Fisher's exact test for categorical variables, and Wilcoxon signed-rank tests for paired samples. Multivariate analysis was performed using a Gamma regression model to evaluate group differences. The model was adjusted for the following covariates: age, sex, weight, ASA score, and the extent of spinal fusion (number of fused vertebrae >10). Missing data were not imputed, and analyses were performed based on available data (available-case analysis). Correlation analyzes were performed according to Spearman. The statistical significance level was set at 0.05. All statistical analyses were performed using available data for each variable (available-case analysis).

## 3 Results

### 3.1 Demographics

Between January 1, 2020, and June 30, 2023, 193 patients underwent surgery for spinal deformity correction. After excluding patients with degenerative scoliosis (n = 47), minors under 15 years of age (n = 6), prior spinal surgeries (n = 3), chronic opioid use (n = 1), and neurologic scoliosis (n = 9), a total of 119 patients were included in the final analysis. Patients were allocated as previously described, based on the analgesic strategy: 52 in *Group IA* (43.7%), 32 in *Group ESPB* (26.9%), 9 in *Group ITM* (7.6%), and 26 in *Group ESPB + ITM* (21.8%). The cohort was predominantly female (80%) with a median age of 21 years (range 15–30) and a median weight of 56 kg (range 35–96), consistent across all groups. Most patients were ASA I (78%), with no cases classified ASA ≥ III. Operative time was similar across groups, with a median surgery duration of 179 min. The median number of fused vertebrae was 12 in the global cohort and similar in the different groups. In our cohort, the distribution of Lenke AIS curve types comprised 57 (47.9%) type 1, 14 (11.8%) type 2, 13 (10.9%) type 3, 20 (16.8%) type 5, and 15 (12.6%) type 6. Overall, the demographic and surgical characteristics were comparable between patient groups (Table 1).

**Table 1:** Main characteristics of the study population and surgical data.

Factors		Total N = 119	Group 1 N = 52	Group 2 N = 32	Group 3 N = 9	Group 4 N = 26
***Patient***						
Age (years)	Median (IQR)	21 (18–29)	22 (19–26)	21 (18–29)	21 (17–24)	20 (18–28)
	Range	15–30	15–30	15–30	15–29	15–29
Weight (kg)	Median (IQR)	56 (52–66)	55 (52–65)	59 (53–65)	60 (50–64)	58 (53–66)
	Range	35–96	44–96	41–89	35–93	44–86
BMI (kg/m^2^)	Median (IQR)	20.6 (18.4–23.7)	20.1 (18.8–23.5)	20.7 (18.8–23.3)	18.5 (17.9–22.7)	21.2 (17.9–24.5)
	Range	15.1–37.5	15.8–37.5	16.6–30.8	15.1–36.2	16.4–28.7
Gender	Female	95 (80%)	47 (90%)	24 (75%)	7 (78%)	17 (65%)
	Male	24 (20%)	5 (9.6%)	8 (25%)	2 (22%)	9 (35%)
ASA score	I	93 (78%)	47 (90%)	18 (56%)	8 (89%)	20 (77%)
	II	26 (22%)	5 (9.6%)	14 (44%)	1 (11%)	6 (23%)
***Surgery***						
Number of instrumented levels	Median (IQR)	12 (10–13)	12 (11–13)	12 (11–12.25)	8 (7–12)	11.5 (8–13)
	Range	6–15	7–15	7–14	6–14	6–15
Operative time (min)	Median (IQR)	179 (156–201)	184 (159–207)	179 (156–191)	166 (136–182)	179 (162–201)
	Range	109–291	121–250	118–254	118–218	109–291

(kg = kilograms, m = meters, min = minutes, IQR = Inter-Quartile Range).

### 3.2 Postoperative morphine consumption

**Fig. 2:**
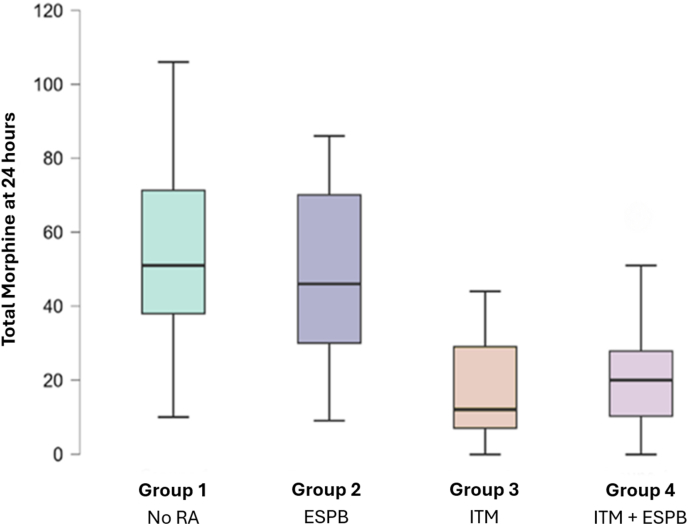
Morphine consumption at 24 h according to the perioperative analgesic strategy. The medians, interquartile ranges, and extreme values are shown. (RA = Regional Anesthesia).

Table 2 demonstrates postoperative morphine consumption, expressed as intravenous (IV) morphine milligram equivalent In *Group IA* the morphine consumption was 51 mg (38–71), and 46 mg (30–70) in *Group ESPB*. The addition of ITM reduced postoperative morphine consumption, with a total dose of 12 mg (7–29) in *Group ITM* and 20 mg (10–28) in *Group ESPB + ITM*, both reductions being significant (p < 0.001) compared to IA and ESPB. These results and their distribution are illustrated by box plots in Fig. 2.

**Table 2:** Morphine consumption by group over the first three postoperative days.

Factors	Total N = 119	Group 1 N = 52	Group 2 N = 32	Group 3 N = 9	Group 4 N = 26	p-value^1^
***Total morphine equivalent at 24 h***						***<0.001***
Median (IQR)	39 (22–63)	51 (38–71)	46 (30–70)	12 (7–29)	20 (10–28)	
Range	0–106	10–106	9–86	0–44	0–64	
***Total morphine equivalent at 48 h***						***0.2***
Median (IQR)	43 (21–58)	49 (16–64)	51 (35–58)	35 (23–43)	34 (25–46)	
Range	1–75	3–75	4–74	1–51	2–67	
***Total morphine equivalent at 72 h***						***<0.001***
Median (IQR)	82 (51–111)	102 (81–122)	86 (66–111)	46 (23–67)	46 (21–65)	
Range	0–190	20–190	12–143	0–72	7–102	

(IQR = Inter-Quartile Range,**^1^** = Kruskal-Wallis rank sum test).

To reduce the effect of extreme body weights, we also analyzed morphine consumption per kilogram of actual body weight. Again, *Groups ITM* and *ESPB + ITM* showed a significant reduction in morphine consumption, with decreases of −0.65 mg/kg and −0.58 mg/kg respectively. A gamma regression model showed a significant 67.1% reduction in morphine in the *ITM group*, OR = 0.32, 95% CI [0.23; 0.49], while the *ESPB group* showed a non-significant 12.6% reduction.

**Fig. 3:**
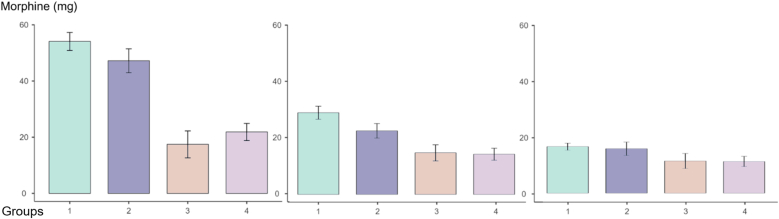
Histogram of daily morphine consumption over 72 postoperative hours by group, in IV morphine equivalents (mg).

At 48 h, morphine consumption did not differ significantly between groups (p = 0.20), though cumulative 72-h use remained lower in ITM groups (Table 2). At 72 h, patients with ITM (*Groups ITM and ESPB + ITM*) consumed a median of 46 mg of morphine, compared to 86 mg in *Group ESPB* and 102 mg in *Group IA* (p < 0.001). On day 3, consumption decreased in all groups, with no rebound effect. Patients receiving ITM maintained a stable consumption of less than 20 mg per day (Fig. 3).

For patients with more than 10 vertebrae fused, the morphine dose was multiplied by 1.28 compared to those with less than 10 levels fused, representing an increase of about 4.4% of morphine for each additional vertebra (p = 0.045).

Multivariate analysis confirmed that ITM was the only significant (p < 0.001) factor, with a consistent and robust 57.9% reduction in postoperative morphine use compared to others, without confounding factors.

### 3.3 Analgesic drugs

The detailed analysis of analgesic medications during general anesthesia is demonstrated in Table 3. In patients who received ESPB and/or ITM, a lower median sufentanil dose was used compared to *Group IA* during the surgical procedure.

**Table 3:** Analgesic drugs according per group.

Factors		Total N = 119	Group 1 N = 52	Group 2 N = 32	Group 3 N = 9	Group 4 N = 26	p-value^1^
***Analgesia***							
Sufentanil (μg)	Median (IQR)	35 (30–45)	45 (35–54)	35 (25–40)	35 (35–40)	30 (21–39)	<0.001
Ketamine (mg)	Median (IQR)	30 (18–33)	20 (10–30)	30 (28–40)	15 (10–20)	30 (21–40)	0.002
Clonidine	n (%)	61 (51%)	18 (35%)	24 (75%)	6 (67%)	13 (50%)	0.003
Magnesium	n (%)	86 (72%)	30 (58%)	30 (94%)	8 (89%)	18 (69%)	0.001
Dexamethasone	n (%)	108 (91%)	43 (83%)	32 (100%)	8 (89%)	25 (96%)	0.026
Lidocaine	n (%)	77 (65%)	46 (88%)	11 (34%)	8 (89%)	12 (46%)	<0.001

(μg = micrograms, mg = milligrams, IQR = Inter-Quartile Range, **^1^** = Kruskal-Wallis rank sum test).

### 3.4 Postoperative pain

The VAS revealed a significant (p < 0.001) reduction of pain scores in groups using ITM (*ITM and ESPB + ITM*), with a minimum decrease of 2 points in the first 24 postoperative hours. This effect persisted until day 3 after surgery (Table 4).

**Table 4:** Postoperative Visual Analog Scale (VAS) scores by group.

VAS Scores	Total N = 119	Group 1 N = 52	Group 2 N = 32	Group 3 N = 9	Group 4 N = 26	p-value^1^
***Initial in PACU***						***< 0.001***
Median (IQR)	2 (0–5)	3 (0–6)	3.5 (0–4.25)	0 (0–5)	0 (0–0)	
Range	0–10	0–10	0–10	0–7	0–8	
***Worst in PACU***						***< 0.001***
Median (IQR)	5 (3–7)	5 (3–7)	6 (5–8)	3 (0–5)	2.5 (1–4)	
Range	0–10	0–10	2–10	0–7	0–8	
***Best at 24h***						***0,001***
Median (IQR)	2 (2–3)	3 (2–4)	2 (2–3)	2 (0–2)	2 (0–2)	
Range	0–7	0–7	0–5	0–5	0–6	
***Worst at 48h***						***0.001***
Median (IQR)	6 (4–7)	6 (5–8)	6 (5–7)	4 (4–5)	5 (4–6)	
Range	0–10	3–10	3–10	2–6	0–8	
***Worst at 72h***						***0.005***
Median (IQR)	6 (4–7)	6 (5–7)	6 (4–7)	3 (2–5)	5 (3–6.75)	
Range	0–10	2–9	2–10	0–6	0–8	

(VAS = Postoperative Visual Analog Scale, PACU = Post-Anesthesia Care Unit, IQR = Inter-Quartile Range,**^1^** = Kruskal-Wallis rank sum test).

### 3.5 Recovery and adverse events

There were no significant differences between groups regarding perioperative bleeding, transfusion requirements, or HemoCue® variations. Opioid and local anesthetic-related side effects such as postoperative nausea and vomiting, spontaneous return of urination or bowel function were also similar across groups. No cases of post-dural puncture syndrome or systemic local anesthetic toxicity were reported. Recovery metrics, including time to first mobilization and length of hospital stay, were comparable across all groups. Detailed recovery outcomes are presented in Table 5.

**Table 5:** Postoperative recovery outcomes by group.

	Total N = 119	Group 1 N = 52	Group 2 N = 32	Group 3 N = 9	Group 4 N = 26	p-value^1^
**Time to first mobilization (days)**						***0.07***
Median (IQR)	1 (1–1.25)	1 (1–2)	1 (1–1)	2 (1–2)	1 (1–1)	
Range	1–3	1–3	1–3	1–3	1–3	
**Time to urinary catheter removal (days)**						***0.11***
Median (IQR)	3 (3–4)	3 (3–4)	3 (2–4)	4 (3–5)	3 (3–4)	
Range	1–7	2–7	1–7	2–6	1–5	
**Return of bowel function by Day 4**						***> 0.9***
No	96 (81%)	43 (83%)	25 (78%)	7 (78%)	21 (81%)	
Yes	23 (19%)	9 (17%)	7 (22%)	2 (22%)	5 (19%)	
**Length of hospital stay (days)**						***0.28***
Median (IQR)	7 (6–8)	7 (6–8)	7 (6–7.25)	8 (7–8)	6.5 (6–7)	
Range	4–11	4–11	4–9	4–9	4–10	

(Values are expressed as median (IQR) or count (%). P-values**^1^** calculated using Kruskal-Wallis test or Fisher's exact test.).

Oxygen requirement at 24 h was significantly lower in *ITM groups* (median 0 L/min [0–1]) compared to the *IA group* (median 1 L/min [0–1]) (p < 0.001). Regarding potential confounders, the cohort consisted of young adults without major comorbidities such as restrictive ventilatory disorder (ASA I-II). We observed a weak correlation between intraoperative sufentanil dose and postoperative oxygen requirement (rho = 0.28, p = 0.002). Since sufentanil consumption was significantly lower in *ITM groups* (p < 0.001), this finding is consistent with the protocol's opioid-sparing effect.

## 4 Discussion

Multimodal perioperative analgesia including RA as part of ERAS principle in AIS surgery can accelerate convalescence and decrease perioperative morbidity (Pico et al., 2022; Brigato et al., 2025). Postoperative systemic morphine consumption is associated with dose-dependent side effects that inhibit early mobilization and fast recovery, unlike RA techniques (Memtsoudis et al., 2018). The use of ESPB and ITM is intended to optimize analgesia and reduce postoperative morphine use as part of ERAS. The SAPOS study was designed to determine the influence of each analgetic method.

The use of ESPB has expanded over the past decade in spine surgery, particularly for short-level procedures (Muthu et al., 2024). This technique is relatively easy to perform, reproducible and safe, which contributed to its large adoption in various situations. However, our study focused on major posterior spinal fusions, where ESPB didn't significantly reduce 24 h postoperative morphine consumption. This limited effect is consistent with different studies by Zhang et al. (2021a) and Zhang et al. (2021b). They thus observed short-term benefits (mainly within the first 12 postoperative hours), but no significant impact at 24 h, both in terms of pain reduction and morphine consumption. Zhu et al. (2021) reported reduced oxycodone consumption over 48 h when using an ESPB in mono- or bi-segmental lumbar fusion surgery. A recent meta-analysis showed decreased pain scores, morphine use, and postoperative nausea and vomiting incidence (Liang et al., 2021). However, these findings are limited by heterogeneous surgical procedures and small number of spinal levels operated. A 2023 Cochrane review concluded that ESPB may slightly reduce pain at 24 h, but without clinically meaningful impact (Schnabel et al., n.d.). The lack of significant benefit from ESPB observed in our study can be analyzed through four distinct factors. First, regarding technical limitations in extensive surgery, the analgesic coverage of ESPB (known to have a restricted cranio-caudal spread (Ivanusic et al., 2018)) likely fails to cover the massive nociceptive field of major deformity correction (median 12 levels) compared to short-segment surgery. Second, regarding dosing variability, our protocol utilized lower concentrations (0.1%–0.2%) to remain within safety limits regarding local anesthetic toxicity. This likely resulted in an insufficient ‘dose per vertebral level' to provide a dense sensory block. Third, this disparity in surgical extent and dosing explains the inconsistent literature. For instance, Zhu et al. (2021) reported efficacy using higher concentrations (0.375%) in mono- or bi-segmental fusions, a regimen that is difficult to scale to extensive multilevel corrections. Finally, the variability in injection techniques inherent to the retrospective design highlights the critical need for standardized protocols in future prospective trials to optimize volume and concentration ratios.

ITM is recognized as an effective technique for pain management in several meta-analyses and is currently recommended as a first line option for some procedures, particularly in visceral, gynecologic and orthopedic surgeries using doses ranging from 50 to 200 μg (Roofthooft et al., 2021; Bang et al., 2023; Lavand'homme et al., 2022). A recent meta-analysis evaluated the use of ITM for degenerative spine surgery in four different studies, with higher doses of morphine (from 200 to 400 μg) (Geisler et al., 2022). It reports improved pain scores at 6 and 24 h postoperative, without adverse events. However, the evidence is limited by large heterogeneity and methodological bias across studies. Although the range of morphine doses described in published studies is wide, we decided to elaborate our local protocol with lower dosage, based on what general and obstetric fields are used to, meaning 100 μg of morphine. It provides similar efficacy with no noticeable adverse events and no need for postoperative monitoring in an intensive care or step-down unit. Our findings showing that ITM might be more effective than ESPB in terms of analgesia and opioid-sparing, are consistent with recent prospective studies performed in spine surgery (Mahmoud et al., 2023; Lal et al., 2024). The observed superiority of ITM must be interpreted considering the fewer instrumented levels in the ITM-only group, which may independently reduce pain and opioid needs. Residual confounding may overestimate ITM's true effect.

Combining ITM and ESPB would be supposedly interesting due to their pharmacological distinct and potentially synergistic mechanisms. However, we didn't show better outcomes when associating ITM with ESPB. We established that morphine consumption was lower with ITM alone, although this may be biased by a smaller sample size and less extensive vertebral levels. Pain scores and adverse events were similar, and the slight reduction of peroperative sufentanil dose remains of unclear clinical significance. Other studies in different surgery using ITM with 100 μg of morphine showed similar results like the present study, with a reduction of about 20 mg of morphine consumption during the first twenty-four post-operative hours, and a reduction of 1–2 points of pain scores over 48 h (Bang et al., 2023; Pitre et al., 2020). This goes along with the needs of patients operated on posterior spinal fusion, who typically experience severe pain in the immediate postoperative period and adequate pain relief can enable mobilization from day one (Kwan et al., 2017). Finally, a recent narrative review (Reysner et al., 2025) corroborates our findings in terms of effectiveness of ITM but is at variance with our results about ESPB. Thus, ESPB is a technique that needs further investigation, particularly in extensive surgeries with multilevel approach.

In our study, the population was quite homogenous, and the surgical approach standardized with techniques used by the same team. Most patients were mobilized between the first and second postoperative day, with no significant group differences. Significantly lower oxygen requirements in ITM patients likely reflect reduced respiratory depression and better-preserved respiratory mechanics. Early oxygen weaning improves comfort and facilitates mobilization by removing physical constraints. The sample sizes in subgroups were limited because of the retrospective and non-randomized nature of the study, which prevented the analysis of confounding factors regarding scoliosis severity, surgical instrumentation and reduction techniques. The single-center design is also a major constraint. Moreover, the choice of anesthetic technique was left to the anesthesiologist, according to his experience and preferences, and IV lidocaine was occasionally used in the *IA group*, introducing further variability. Furthermore, the ITM-only group had a median of 8 instrumented levels compared to 12 in other groups, which may have contributed to lower pain scores observed in this subgroup. Additionally, the use of intravenous lidocaine was more frequent in the IA group (88%) compared to others, introducing a confounding factor by indication.

## 5 Conclusion

In this retrospective cohort, ITM (median dose 100 μg) was associated with significantly reduced opioid use and improved pain scores, with no noticeable side effects observed. Conversely, ESPB did not show a significant additive effect. Based on these results, ITM is now routinely used in our ERAS protocol in spinal deformity surgery. However, prospective randomized trials are needed to confirm these findings and optimize regional analgesic techniques in AIS surgery.

## Ethics approval

Institutional review board approval was obtained in 2023 under the reference CE-2023-62 and declared on ClinicalTrials.gov ID NCT06194279.

## Use of AI tools

None.

## Funding

None.

## Conflicts of interest unrelated to this study

Yann Philippe Charles is consultant for Stryker, Clariance.
