# Supplementary material for: Comparative effectiveness of intrathecal morphine versus erector spinae plane block for analgesia after scoliosis surgery: a retrospective analysis
Source: Brain Spine. 2026 Feb 2;6:105957. doi: 10.1016/j.bas.2026.105957 (PMC12892043; doi:10.1016/j.bas.2026.105957)
Supplement: Multimedia component 1 [file mmc1.docx]

STROBE Statement—checklist of items that should be included in reports of observational studies

|  | Item No. | Recommendation | Page  No. | Relevant text from manuscript |
| --- | --- | --- | --- | --- |
| **Title and abstract** | 1 | (*a*) Indicate the study’s design with a commonly used term in the title or the abstract | 1 | Comparative effectiveness of intrathecal morphine versus erector spinae plane block for analgesia after scoliosis surgery: a retrospective analysis |
|  |  | (*b*) Provide in the abstract an informative and balanced summary of what was done and what was found | Abstract file | Intrathecal morphine injection during scoliosis surgery reduced morphine consumption and postoperative pain scores… |
| Introduction | | | |  |
| Background/rationale | 2 | Explain the scientific background and rationale for the investigation being reported | 1 | Adolescent idiopathic scoliosis (AIS) correction and fusion are extensive procedures that require effective pain management... Multimodal analgesia is recommended when following Enhanced Recovery After Surgery (ERAS) principles |
| Objectives | 3 | State specific objectives, including any prespecified hypotheses | 2 | The purpose of this study was to analyze the analgesic efficacy of ESPB and ITM while determining the role of each technique in lowering 24h postoperative morphine consumption as primary outcome parameter. |
| Methods | | | |  |
| Study design | 4 | Present key elements of study design early in the paper | 2 | The Scoliosis Analgesia Per-Operative Strategies (SAPOS) study was a retrospective, monocentric, and non-interventional study... |
| Setting | 5 | Describe the setting, locations, and relevant dates, including periods of recruitment, exposure, follow-up, and data collection | 2 | ...conducted to compare different perioperative analgesic strategies in scoliosis surgery... between January 1, 2020, and June 30, 2023 |
| Participants | 6 | (*a*) *Cohort study*—Give the eligibility criteria, and the sources and methods of selection of participants. Describe methods of follow-up  *Case-control study*—Give the eligibility criteria, and the sources and methods of case ascertainment and control selection. Give the rationale for the choice of cases and controls  *Cross-sectional study*—Give the eligibility criteria, and the sources and methods of selection of participants | 2 | All patients over 15 years of age who underwent surgical correction of idiopathic scoliosis were included. Exclusion criteria were previous history of spinal surgery... daily use of opioids... |
|  |  | (*b*) *Cohort study*—For matched studies, give matching criteria and number of exposed and unexposed  *Case-control study*—For matched studies, give matching criteria and the number of controls per case |  |  |
| Variables | 7 | Clearly define all outcomes, exposures, predictors, potential confounders, and effect modifiers. Give diagnostic criteria, if applicable | 3 | The primary outcome measure was total morphine consumption during the first 24 postoperative hours... Secondary outcomes included morphine consumption at 48 and 72 hours… |
| Data sources/ measurement | 8* | For each variable of interest, give sources of data and details of methods of assessment (measurement). Describe comparability of assessment methods if there is more than one group | *2* | *The study analyzed medical records... All doses were standardized into intravenous morphine equivalents...* |
| Bias | 9 | Describe any efforts to address potential sources of bias | 4 | Multivariate analysis included logistic regression models to evaluate group differences and potential confounding factors. |
| Study size | 10 | Explain how the study size was arrived at | 2 | Patients were included using a consecutive sampling method. |

| Quantitative variables | 11 | Explain how quantitative variables were handled in the analyses. If applicable, describe which groupings were chosen and why | 4 | Continuous variables were expressed as medians with interquartile ranges, while categorical variables were presented as counts and percentages. |
| --- | --- | --- | --- | --- |
| Statistical methods | 12 | (*a*) Describe all statistical methods, including those used to control for confounding | 4 | Multivariate analysis was performed using a Gamma regression model to evaluate group differences. The model was adjusted for the following covariates: age, sex, weight, ASA score, and the extent of spinal fusion (number of fused vertebrae >10) |
|  |  | (*b*) Describe any methods used to examine subgroups and interactions | 6 | To reduce the effect of extreme body weights, we also analyzed morphine consumption per kilogram...  For patients with more than 10 vertebrae fused, the morphine dose was multiplied by 1.28… |
|  |  | (*c*) Explain how missing data were addressed | 4 | Missing data were not imputed. All statistical analyses were performed using available data for each variable (available-case analysis). |
|  |  | (*d*) *Cohort study*—If applicable, explain how loss to follow-up was addressed  *Case-control study*—If applicable, explain how matching of cases and controls was addressed  *Cross-sectional study*—If applicable, describe analytical methods taking account of sampling strategy |  | N/A (Retrospective study with in-hospital primary endpoints, no long-term follow-up) |
|  |  | (*e*) Describe any sensitivity analyses | 6 | A gamma regression model showed a significant 67.1% reduction in morphine in the ITM group... while the ESPB group showed a non-significant 12.6% reduction. |
| Results | | | | |
| Participants | 13* | (a) Report numbers of individuals at each stage of study—eg numbers potentially eligible, examined for eligibility, confirmed eligible, included in the study, completing follow-up, and analysed | 5 | Between January 1, 2020, and June 30, 2023, 193 patients underwent surgery... a total of 119 patients were included in the final analysis. Patients were allocated... 52 in Group IA... 32 in Group ESPB... 9 in Group ITM... and 26 in Group ESPB + ITM |
|  |  | (b) Give reasons for non-participation at each stage | 5 | After excluding patients with degenerative scoliosis (n=47), minors under 15 years of age (n=6), prior spinal surgeries (n=3), chronic opioid use (n=1), and neurologic scoliosis (n=9)... |
|  |  | (c) Consider use of a flow diagram |  | N/A |
| Descriptive data | 14* | (a) Give characteristics of study participants (eg demographic, clinical, social) and information on exposures and potential confounders | 5 | The cohort was predominantly female (80%) with a median age of 21 years... and a median weight of 56 kg... Most patients were ASA I (78%)... See Table 1. |
|  |  | (b) Indicate number of participants with missing data for each variable of interest | 5 | Overall, the demographic and surgical characteristics were comparable between patient groups (Table 1). |
|  |  | (c) *Cohort study*—Summarise follow-up time (eg, average and total amount) | 3, 7 | Secondary outcomes included morphine consumption at 48 and 72 hours postoperatively... The timing of return to normal diet, spontaneous micturition, time to first mobilization, and length of hospital stay were comparable. |
| Outcome data | 15* | *Cohort study*—Report numbers of outcome events or summary measures over time | 5 | Table 2 demonstrates postoperative morphine consumption... In Group IA the morphine consumption was 51 mg (38-71)... The addition of ITM reduced postoperative morphine consumption, with a total dose of 12 mg (7-29) in Group ITM... |
|  |  | *Case-control study—*Report numbers in each exposure category, or summary measures of exposure |  |  |
|  |  | *Cross-sectional study—*Report numbers of outcome events or summary measures |  |  |
| Main results | 16 | (*a*) Give unadjusted estimates and, if applicable, confounder-adjusted estimates and their precision (eg, 95% confidence interval). Make clear which confounders were adjusted for and why they were included | 5,6 | In Group IA the morphine consumption was 51 mg (38-71)... total dose of 12 mg (7-29) in Group ITM... (p<0.001).  A gamma regression model showed a significant 67.1% reduction in morphine in the ITM group (OR coefficient = 0.32, 95% CI [0.23: 0.49]) ... Multivariate analysis confirmed that ITM was the only significant (p<0.001) factor. |
|  |  | (*b*) Report category boundaries when continuous variables were categorized | 6 | For patients with more than 10 vertebrae fused, the morphine dose was multiplied by 1.28 compared to those with less than 10 levels fused... |
|  |  | (*c*) If relevant, consider translating estimates of relative risk into absolute risk for a meaningful time period |  | N/A |

| Other analyses | 17 | Report other analyses done—eg analyses of subgroups and interactions, and sensitivity analyses | 6 | To reduce the effect of extreme body weights, we also analyzed morphine consumption per kilogram...  For patients with more than 10 vertebrae fused, the morphine dose was multiplied by 1.28... |
| --- | --- | --- | --- | --- |

| Discussion | | | | |
| --- | --- | --- | --- | --- |
| Key results | 18 | Summarise key results with reference to study objectives | 9 | Our findings showing that ITM might be more effective than ESPB in terms of analgesia and opioid-sparing, are consistent with recent prospective studies performed in spine surgery. |
| Limitations | 19 | Discuss limitations of the study, taking into account sources of potential bias or imprecision. Discuss both direction and magnitude of any potential bias | 10 | However, the sample sizes in subgroups were limited because of the retrospective nature of the study... The single-center design is also a major constraint. Moreover, the choice of anesthetic technique was left to the anesthesiologist... and IV lidocaine was occasionally used... introducing further variability.  Furthermore, the ITM-only group had a median of 8 instrumented levels compared to 12 in other groups... Additionally, the use of intravenous lidocaine was more frequent in the IA group (88%) compared to others, introducing a confounding factor by indication |
| Interpretation | 20 | Give a cautious overall interpretation of results considering objectives, limitations, multiplicity of analyses, results from similar studies, and other relevant evidence | 8,9 | The limited efficacy of ESPB observed in our study, is probably linked to the large posterior approach with a median of 12 instrumented vertebrae...  ITM is recognized as an effective technique... and is currently recommended as a first line option... |
| Generalisability | 21 | Discuss the generalisability (external validity) of the study results | 10 | In our study, the population was quite homogenous, and the surgical approach standardized with techniques used by the same team... ITM is now routinely used in our ERAS protocol... |
| Other information | |  | | |
| Funding | 22 | Give the source of funding and the role of the funders for the present study and, if applicable, for the original study on which the present article is based |  | No funding was received for this study. |
